# Clinicopathological characteristics of neural epidermal growth factor-like 1 protein-associated membranous glomerulonephritis

**DOI:** 10.1007/s00428-024-03921-6

**Published:** 2024-09-09

**Authors:** Toshiki Hyodo, Shigeo Hara, Shunsuke Goto, Hideki Fujii, Shinichi Nishi, Akihiro Yoshimoto, Tomoo Itoh

**Affiliations:** 1 Department of Diagnostic Pathology, Kobe University Graduate School of Medicine, Kobe, Japan; 2 Department of Diagnostic Pathology, Kobe City Medical Center General Hospital, Kobe, Japan; 3 Division of Nephrology, Kobe University Graduate School of Medicine, Kobe, Japan; 4 Department of Nephrology, Kobe City Medical Center General Hospital, Kobe, Japan

**Keywords:** Membranous glomerulonephritis (MGN), Neural epidermal growth factor-like 1 protein (NELL1), Bucillamine, Segmental MGN, IgG1-dominant IgG subclass

## Abstract

Neural epidermal growth factor-like 1 protein (NELL1) is the second most common target antigen in membranous glomerulonephritis (MGN). However, data regarding the clinicopathological characteristics of NELL1-associated MGN are limited owing to its low prevalence. This study examined the prevalence and clinicopathological characteristics of NELL1-associated MGN in a Japanese cohort. Additionally, we compared the clinicopathological features of NELL1-positive MGN, phospholipase A2 receptor 1 (PLA2R1)-positive MGN, and MGN negative for all three antigens (NELL1, PLA2R1, and thrombospondin type-1 domain-containing 7A). Among 257 consecutive patients pathologically diagnosed with MGN at two centers in Japan, 24 (9.3%) were immunohistochemically positive for NELL1. Clinically, patients with NELL1-positive MGN were significantly older (*p* < 0.001) and had a higher frequency of bucillamine use (vs PLA2R1-positive MGN, *p* < 0.01). Histologically, NELL1-positive MGN exhibited significantly lower detection of spikes and crater formation (*p* < 0.001), higher prevalence of segmental spike distribution (vs PLA2R1-positive MGN: *p* < 0.001), and higher prevalence of stage I cases on electron microscopy (*p* < 0.01). There were no significant differences in the prognoses among the three groups. The characteristic histological feature of segmental distribution in NELL1-positive MGN may be related to bucillamine use and the early phase of the disease. Further investigations with larger numbers of patients may offer further insight into the prognosis of patients with NELL1-positive MGN.

## Introduction

Histopathologically, membranous glomerulonephritis (MGN) is characterized by subepithelial deposition of immune complexes in the glomerular basement membrane (GBM) and is the most common cause of nephrotic syndrome in adults [1]. Etiologically, MGN has been classified as a primary (idiopathic) disease caused by autoantibodies binding to specific target antigens, as well as a secondary disease, which is associated with a variety of systemic factors including autoimmune diseases, neoplasms, infections, and drugs [1, 2]. This binary categorization has been recently replaced by the classification that focused on the target-antigen based approach. In 2009, an antibody specific to phospholipase A2 receptor 1 (PLA2R1), which is an antigen endogenous to podocytes, was observed to be positive in 70–80% of idiopathic MGN cases [3]. Subsequently, thrombospondin type-1 domain-containing 7A (THSD7A) [4], exostosin 1/2 (EXT1/2) [5], and neural epidermal growth factor-like 1 protein (NELL1) [6] were identified as causative antigens in MGN. EXT1/2-associated MGN is regarded as secondary MGN as it is related to autoimmune diseases such as membranous lupus nephritis. The remaining primary causative antigens comprise approximately 80–90% of primary MGN cases [2, 7, 8].

NELL1-associated MGN was discovered by Sethi et al. who observed this phenotype in 16% of PLA2R1-negative MGN cases [6]. Subsequent studies have shown that the frequency of NELL1 positivity was 2–2.5% among primary MGN cases [7, 8]. According to research conducted more recently, the NELL1 antigen accounts for 10%, making it the second most common antigen [2]. The NELL1 positivity rate was as high as 33% (30/91) in patients with MGN complicated by malignancy, indicating a possible association with malignancy-related MGN [9]. More recent reports have shown that NELL1-associated MGN is associated with lipoic acid use [10, 11], traditional indigenous medicine (mercury) exposure [12], hematopoietic stem cell transplantation [13], and autoimmune diseases including rheumatoid arthritis (RA) [14]. On immunofluorescence (IF), NELL1-associated MGN often exhibits segmental IgG positivity and IgG1-dominant IgG subclass [9]. These features differ from those of other types of primary MGNs that exhibit global IgG positivity and an IgG4-dominant IgG subclass.

In Japan, the incidences of autoantigen-associated MGNs differ from those in other countries [15]. For instance, in Japan, the frequency of PLA2R1-associated MGN is approximately 60% among primary MGNs [15, 16], in contrast to the higher rate of 70–80% found in Western countries [3, 17], China [18], and Korea [19]. Additionally, in Japan, the rate of THSD7A-associated MGN is 9.1% (5/55) [20], which is higher than that in other countries　[21, 22]. The frequencies of PLA2R1-, THSD7A-, and NELL1-associated MGNs among 69 Japanese patients with primary MGN were 53.6%, 8.7%, and 1.5% [23]. These differences may indicate distinct etiologies of MGN in the Japanese population. Therefore, we herein investigated the incidence and clinicopathological features of NELL1-associated MGN in Japanese patients.

## Materials and methods

### Patients

The present study enrolled patients with MGN, including membranous lupus nephritis, pathologically confirmed by renal biopsy at the Kobe University Hospital and Kobe City Medical Center General Hospital between January 1, 1991, and December 31, 2021. NELL1 immunohistochemical staining was performed for all available specimens to determine the rate of NELL1 positivity among all MGN cases. Immunostaining for PLA2R1 and THSD7A was performed in all NELL1-positive cases and some NELL1-negative cases. The results were divided into positive and negative groups for each antigen. Clinical and pathological factors of NELL1 single-positive cases were compared with those of PLA2R1 single-positive and triple antigen-negative (Negative) MGN cases. Additionally, we referred to the data of patients assessed in our previous studies [16, 24]. This study was approved by the ethics committees of Kobe University Hospital (B210194) and Kobe City Medical Center General Hospital (21267) and was conducted in accordance with the 1975 Declaration of Helsinki.

### Materials

**Fig. 1:**
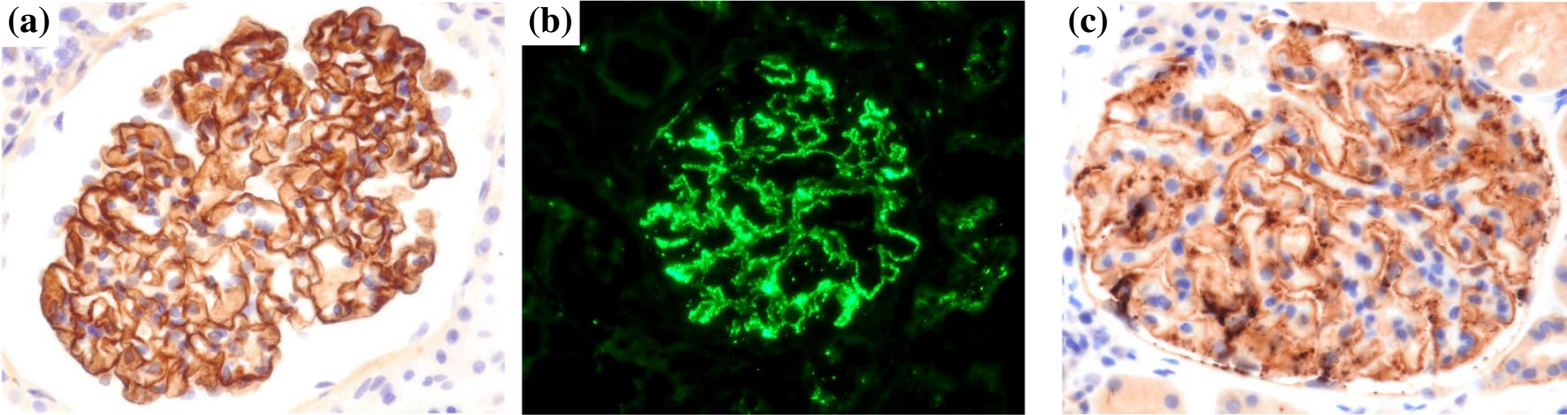
Representative images demonstrating antigen positivity. Representative images showing the results of (**a**) NELL1, (**b**) PLA2R1, and (**c**) THSD7A immunostaining. (**a**) and (**c**) shows immunohistochemistry images. (**b**) shows an immunofluorescence image. Magnification, 40x. Abbreviations: NELL1, neural epidermal growth factor-like 1 protein; PLA2R, phospholipase A2 receptor; THSD7A, thrombospondin-type 1 domain-containing 7A

Immunostaining for NELL1 and THSD7A was performed using an automatic immunohistochemical staining system (BOND MAX, Leica Biosystems, Wetzlar, Germany). For PLA2R1 immunostaining, frozen or paraffinized specimens were used, which were subsequently assessed using a fluorescence microscope (BZ-X710, Keyence, Osaka, Japan). Paraffinized sections were cut to 4 µm thickness, deparaffinized, and stained with rabbit polyclonal anti-NELL1 antibody (1:100, ab197315, Abcam, Cambridge, UK), rabbit polyclonal anti-THSD7A antibody (1:800, HPA 000923, ATLAS ANTIBODIES, Stockholm, Sweden), and rabbit polyclonal anti-PLA2R1 antibody (1:50, HPA012657, ATLAS ANTIBODIES, Stockholm, Sweden). Frozen sections were then cut to 2 µm thickness and stained with rabbit polyclonal anti-PLA2R1 antibody (1:100, HPA012657, ATLAS ANTIBODIES, Stockholm, Sweden). Fig. 1 showed these representative positive images.

### Clinical information

Clinical information regarding the following factors at the time of diagnosis was extracted from medical records: age, sex, the presence of neoplasm, lifestyle-related factors (smoking, hypertension, diabetes, and dyslipidemia), hepatitis B/C virus (HBV/HCV) antibody, autoantibodies, edema symptoms, nephrotic syndrome, levels of creatinine (Cr), Japanese Society of Nephrology (JSN)-estimated glomerular filtration rate (eGFR), complement component 3 (C3), total serum protein (TP), Albumin (Alb), and the presence of abnormalities in qualitative hematuria and proteinuria. For proteinuria and hematuria, qualitative tests were defined as 1 + or more positive. Estimated degrees of proteinuria based on g/gCr values and selective index values were also extracted.

To evaluate prognoses, we obtained clinical information and the degree of remission 1 year after renal biopsy. The collected clinical information included levels of Cr, JSN eGFR, TP, and Alb, qualitative/quantitative proteinuria, the presence of thrombosis, and treatment information (non-steroid, steroid only, and steroid with immunosuppressive therapy). Remission was defined based on the Kidney Disease: Improving Global Outcomes 2012 [25] / 2021 [26] Clinical Practice Guideline for the Management of Glomerular Diseases as follows: complete remission as proteinuria < 0.3 g/day, partial remission as proteinuria 0.3–3.5 g/day or > 50% proteinuria reduction, and no remission as otherwise.

### Histological analysis

Light microscopy (LM) was used to evaluate the following factors of periodic acid-Schiff and periodic acid-methenamine silver-stained specimens: the percentage of global sclerotic glomeruli with respect to the total number of glomeruli, degree of glomerular hypertrophy, presence of spike/crater formation, thickness of the mesangial matrix, mesangial proliferation, endocapillary hypercellularity, extracapillary hypercellularity, segmental sclerosis, degree of interstitial fibrosis and tubular atrophy (IFTA), arteriosclerosis, and arteriolar hyalinosis. Regarding spike/crater formation, segmental or global distribution was evaluated. In addition, chronic lesion scores (glomerular sclerosis [GS], IFTA, and arteriosclerosis scores) were calculated [27]. IF staining of complements (C1q and C3) and immunoglobulins (IgG and IgA) was evaluated for positivity (staining intensity of 1 + or greater was considered positive), GBM distribution of IgG positive images (when IgG was negative, other stains were substituted if possible), and predominant IgG subclass were assessed. GBM lesions < 50% were considered segmental, and the most predominant glomerular findings were adopted. For electron microscopy (EM), the Ehrenreich–Churg stage classification was used; in the mixed-stage case, the predominant stage was adopted.

Two pathologists (TH and SH) specializing in nephropathology performed pathological and immunohistological assessments. The Renal Pathology Society (RPS) Working Group consensus definitions for glomerular lesions by LM and EM were used for all evaluations [28].

### Statistical analysis

Statistical analyses were performed using EZR Software (Jichi Medical University, Japan, http://www.jichi.ac.jp/saitama-sct/). Clinical and pathological factors of the NELL1-positive group were compared with those of other groups. When assessing continuous variables, it was unclear whether they followed a normal distribution; therefore, all analyses were performed using Mann–Whitney U test. Fisher's exact test was used to analyze binary variables. A *p*-value < 0.01 was considered statistically significant. As this was an exploratory study, no adjustments for multiplicity were performed.

## Results

**Fig. 2:**
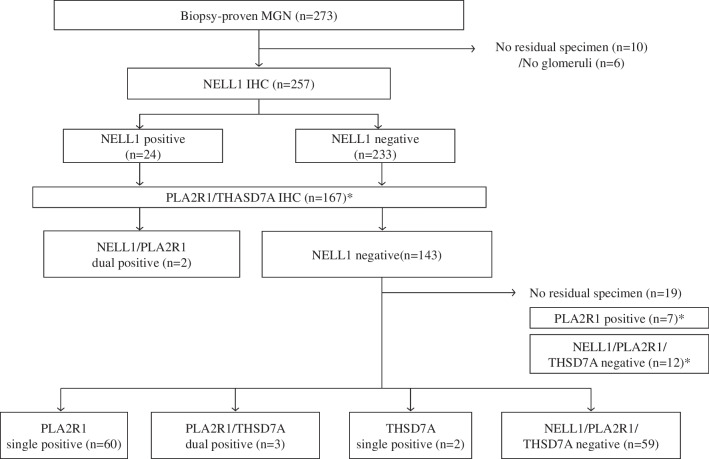
Study design and flowchart of antigen-positive MGN classification. Flowchart of patient enrollment. Cases indicated by asterisks (*) refer to patients included in previous studies ([16, 24]). Abbreviations: IHC, immunohistochemistry; MGN, membranous glomerulonephritis; NELL1, neural epidermal growth factor-like 1 protein; PLA2R, phospholipase A2 receptor; THSD7A, thrombospondin-type 1 domain-containing 7A

In total, 273 patients were diagnosed with MGN during the study period: 136 at the Kobe University Hospital and 137 at the Kobe City Medical Center General Hospital. NELL1 immunostaining was performed in 257 patients, excluding those in whom no residual tissue specimens (*n* = 10) or glomeruli (*n* = 6) were identified (Fig. 2); of these patients, 24 cases (9.3%) were positive for NELL1. All lupus nephritis and secondary MGN cases were negative for NELL1, constituting 10.9% (24/219) of the primary MGN group. PLA2R1 and THSD7A antibody staining was performed in 167 cases, including those from previous studies [16, 24]. The frequencies of PLA2R1-positive and THSD7A-positive cases were 72/167 (43.1%) and 5/167 (3.0%), respectively, including cases with PLA2R1/NELL1 dual positivity (*n* = 2) and PLA2R1/THSD7A dual positivity (*n* = 3). The Negative group (negative for NELL1, PLA2R1, and THSD7A) comprised 71 of the 167 (42.5%) patients.

To delineate the clinicopathological characteristic of NELL1-positive MGN, we compared 22 NELL1 single-positive MGN cases with 60 PLA2R1 single-positive MGN cases and 42 Negative cases, excluding all lupus nephritis cases (*n* = 17); THSD7A single-positive MGN cases were excluded from the comparison because of the small number of cases (*n* = 2) (Fig. 2). Clinically, NELL1-positive MGN was more prevalent in older patients (vs PLA2R1: *p* < 0.001, vs Negative: *p* < 0.001) with a mean age of 74.2 years (range: 58–87, median: 73) of the affected patients (Table 1). Male sex was less prevalent (18.2%) among NELL1-positive MGN cases than among PLA2R1-positive MGN cases (53.3%, *p* < 0.01). Malignancy was detected in 4 (18.2%), 13 (21.7%), and 8 (19%) cases in the NELL1-positive MGN, PLA2R1-positive MGN, and Negative groups, respectively (not significant). Four patients with NELL1-positive MGN were treated with bucillamine (0% among patients with PLA2R1-positive MGN, *p* < 0.01). The mean eGFR in the NELL1-positive MGN group was 62.0 mL/min/1.73 m^2^ (range: 24.1–104, median: 59.8), which was lower than that of the Negative group (78.4 mL/min/1.73 m^2^, range: 13.4–139.9, median: 75.3, *p* < 0.01). The following parameters were not significantly different between the NELL1-positive MGN and other groups: smoking, hypertension, diabetes mellitus, dyslipidemia, HBV/HCV infection, antinuclear antibody positivity, Cr, TP, Alb, C3, edema, proteinuria, nephrotic syndrome, selectivity index, and hematuria.

**Table 1:** Clinical characteristics of NELL1-positive MGN, PLA2R1-positive MGN, and Negative group

	NELL1	PLA2R1	Negative
Number of patients	22	60	42
Age	74.2 [73] (58–87)	61.4 [65.0] (16–84)***	56.3 [61] (16–82)***
Male (%)	4 (18.2)	32 (53.3)**	22 (52.4)*
Malignancy (%)	4 (18.2)	13 (21.7)	8 (19.0)
Bucillamine (%)	4 (18.2)	0**	1 (2.4)*
Lifestyle-related disease (Smoke/Hypertension/Diabetes mellitus/Dyslipidemia)	(5/13/4/17)	(27/35/12/46)	(19/21/9/25)
Infection (HBV/HCV)	(8/3)	(9/0*)	(6/0*)
Antibody positive (ANA/Others)	(3/3)	(19/6)	(19*/9)
Cr (mg/dl)	0.83 [0.76] (0.44–2.21)	0.84 [0.78] (0.27–2.56)	0.88 [0.74] (0.45–3.9)
eGFR (mL/min/1.73m^2^)	62.0 [59.8] (24.1–104)	71.8 [71.5] (19.4–209.8)	78.4 [75.3] (13.4–139.9)**
TP (g/dl)	5.7 [5.8] (4.2–7.6)	5.65 [5.8] (3.9–8.3)	6.14 [6.25] (4.2–9.0)
Alb (g/dl)	2.5 [2.4] (1.2–3.8)	2.74 [2.75] (1.1–4.2)	2.97 [2.95] (1.1–4.9)
C3 (mg/dl)	128 [125] (83–187) (*n* = 18)	116 [113] (69–171) (*n* = 53)	112 [119] (21.2–167) (*n* = 40)
Edema (%)	16 (72.7)	39 (65.0)	12 (50)
Proteinuria (-/ ± /1 + /2 + /3 + /4 +)	(0/0/0/7/6/9)	(0/0/2/13/23/22)	(2/1/0/14/10/15)
Proteinuria (g/gCr)	7.61 [7.0] (1.0–16.3)	6.03 [5.63] (0.19–19.8)	4.83 [2.82] (0.1–21.6) *
Nephrotic syndrome (%)	14 (63.6)	32 (53.3)	21 (50.0)*
Selectivity index	0.40 [0.18] (0.06–3.00) (*n* = 18)	0.41 [0.17] (0.05–4.23) (*n* = 39)	1.33 [0.23] (0.07–8.91) (*n* = 21)
Hematuria (-/ ± /1 + /2 + /3 + /4 +)	(5/4/8/4/1/0)	(6/9/19/19/7/0)	(14/4/9/11/4/0)

Most data value is shown as mean [median] (minimum–maximum). The Mann Whitney U and Fisher's exact tests were used for statistical analysis to compare NELL1-positive MGN to the other groups. (*p < 0.05, **p < 0.01, ***p < 0.001)

**Fig. 3:**
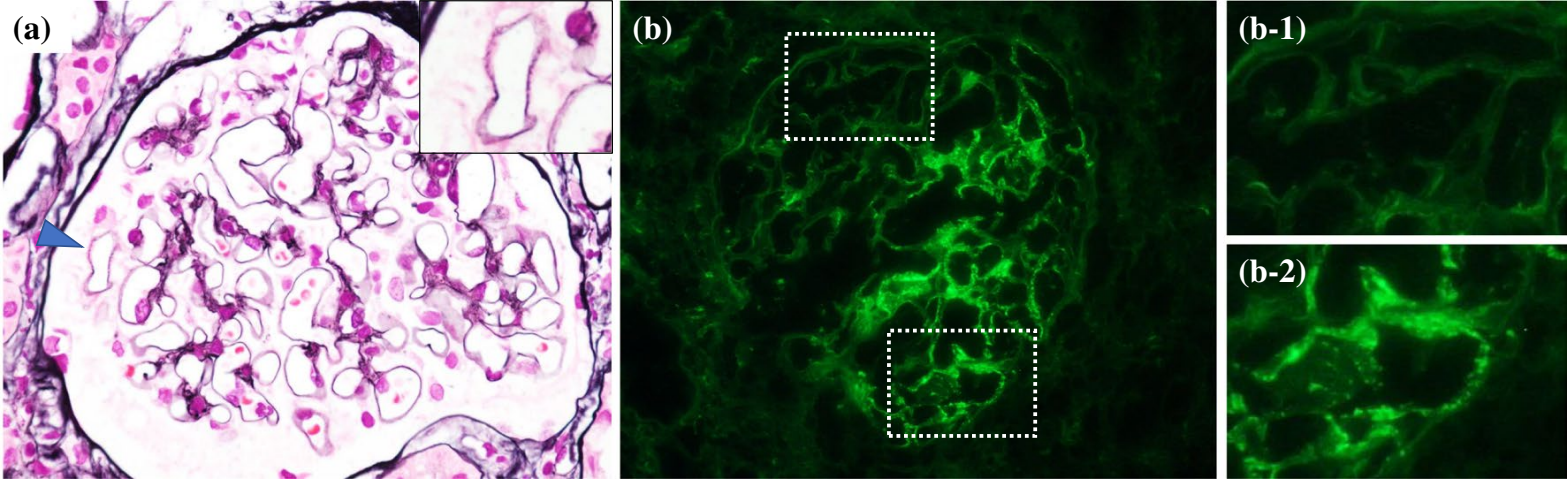
NELL1-positive MGN with segmental distribution of GBM lesions and IgG immunofluorescence. (**a**): Segmental distribution of GBM spikes. Inset shows small spike formations in the segmental part of the GBM. Periodic acid-methenamine silver staining, Magnification, 40x. Inset: Magnification, 80x. (**b**): Segmental IgG immunofluorescence image. Magnification, 40x. Inset indicates (b-1) negative and (b-2) positive granular IgG staining on glomerular capillaries, respectively. Abbreviations: NELL1, neural epidermal growth factor-like 1 protein; MGN, membranous glomerulonephritis; GBM, glomerular basement membrane

Histologically, NELL1-positive MGN cases exhibited a significantly lower prevalence of GBM lesions, such as spikes and crater formation (vs PLA2R1: *p* < 0.001, vs Negative: *p* < 0.001) and a higher percentage of segmental spike distribution (vs PLA2R1: *p* < 0.001) (Fig. 3a, Table 2). Compared with PLA2R1-positive MGN cases, NELL1-positive MGN cases had a higher prevalence of GS score 1 (*p* < 0.001) and IFTA score 1 (*p* < 0.01). A mild grade of chronic changes showed a lower prevalence (*p* < 0.01) in PLA2R1-positive MGN. The degree of glomerular enlargement, proliferative lesions, segmental sclerosis, arteriosclerosis, arteriolar hyalinosis, and the total renal chronicity score were not significantly different between the groups. IF revealed a significantly higher prevalence of segmental IgG positivity in NELL1-positive MGN (Fig. 3b) (vs PLA2R1: *p* < 0.01). The prevalence of IgG/IgA/C1q/C3 positivity and IgG1-dominant and IgG4-dominant immunophenotypes were not significantly different between the groups. EM revealed a significantly higher prevalence of stage I cases (vs PLA2R1: *p* < 0.01, vs Negative: *p* < 0.001) and a significantly lower prevalence of stage III cases (vs Negative: *p* < 0.01) in NELL1-positive MGN. The duration from the onset to the biopsy in NELL1-positive MGN cases were significantly shorter than Negative cases (*p* < 0.01) and the disease stage showed a mild positive correlation (Spearman's rank correlation coefficient 0.29, *p* < 0.01) (data not shown).

**Table 2:** Pathologic characteristics of NELL1–positive MGN, PLA2R1–positive MGN, and Negative group

	NELL1	PLA2R1	Negative
Number of patients	22	60	42
Global sclerotic/total glomeruli^1^	14.3 (0–34.6)	5.5 (0–42.3)*	6.8 (0–77.1)**
GS score (0/1/2/3)^2^	(6/15/1/0)	(34*/16***/8/2)	(23/16*/2/1)
Enlarged (%) > 250µm	4 (18.2)	16 (26.7)	9 (21.4)
Spike/crater (%)	5 (22.7)	48 (80.0)***	35 (83.3)***
Segmental spike distribution (%)	4 (80.0)	3 (6.3)***	9 (25.7)*
Mesangial proliferation (%)	2 (9.1)	17 (28.3)	13 (31.0)
Endocapillary hypercellularity (%)	0	9 (15.0)	3 (7.1)
Extracapillary hypercellularity (%)	0	2 (3.3)	2 (4.8)
Segmental sclerosis (%)	1 (4.5)	13 (21.7)	11 (26.2)*
IFTA score (0/1/2/3)^2^	(7/14/1/0)	(37*/17**/6/0)	(24/15*/1/2)
Arteriosclerosis (0/1)^2^	(5/17)	(21/39)	(20/22)
Arteriolar hyalinosis (no/mild/moderate/severe)	(13/7/2/0)	(35/13/5/7)	(26/12/3/1)
Total renal chronicity score^2^	3.5 (0–5)	1 (0–8)	1.5 (0–10)
Grades of chronic changes^2^ (minimal/mild/moderate/severe)	(4/16/2/0)	(31*/19**/8/2)	(21*/17*/2/2)
IgG positive (%)	21 (95.5)	60 (100)	34/37 (91.9)
Segmental distribution by IF (%)	6/21 (28.6)	1 (1.7)**	2/37 (5.4)*
IgG subclass (IgG1-dominant) (%)	3/9 (33.3)	5/40 (12.5)	5/29 (17.2)
IgG subclass (IgG4-dominant) (%)	5/9 (55.6)	29/40 (72.5)	14/29 (48.3)
C1q positive (over 1 +) (%)	3 (13.6)	16 (26.7)	15/37 (40.5)*
C3 positive (over 1 +) (%)	14 (63.6)	46 (76.7)	25/37 (67.6)
IgA positive (over 1 +) (%)	5 (22.7)	25 (41.7)	20/37 (54.1)*
Ehrenreich-Churg Stage (I/II/III/IV)	(12/4/0/0) (*n* = 16)	(18**/19/16*/2) (*n* = 55)	(6***/18/13**/1) (*n* = 38)

1 Global sclerotic/total glomeruli indicate sclerotic glomerular rate, defined as the percentage of global sclerotic glomeruli in the total number of glomeruli. The data is shown as the median (minimal-maximal) value

2 “GS”, “IFTA”, and Arteriosclerosis score, and “Grades of chronic changes “, and “Grades of chronic changes” are defined by Sechi et al. [28]. “Grades of chronic changes” indicate the grade based on the total renal chronicity score

The Mann Whitney U and Fisher's exact tests were used for statistical analysis to compare NELL1-positive MGN to the other groups. (*p < 0.05, **p < 0.01, ***p < 0.001)

We conducted a comparative study of treatment and clinical prognosis 1 year after renal biopsy (Table 3). Of the eligible patients, 20 were NELL1-positive, 43 were PLA2R1-positive, and 33 were negative. All patients underwent similar treatment. In the NELL1-positive MGN group, eGFR and proteinuria levels were lower than those in the other two groups; however, the differences were not significant. Other clinical parameters including remission rate, serum Cr, TP, Alb, and thrombosis events were not significantly different between the groups.

**Table 3:** Comparison of clinical prognosis in the time of 1 year after renal biopsy among patients with NELL1–positive, PLA2R1–positive, and Negative MGN

	NELL1	PLA2R1	Negative
Number of patients	20	43	33
Medication treatment (Non-steroid/Steroid only/ Steroid + immunosuppressive agent/unknown)	(10/7/2/1)	(17/17^##^/9/0*)	(13^#^/11/9/0*)
Remission (complete/partial/No)	(11/6/3)	(13/24/6)	(13/14/6)
Cr (mg/dl)	0.75 [0.72] (0.41–1.19)	0.86 [0.81] (0.4–1.93)	0.84 [0.73] (0.4–1.93)
eGFR (mL/min/1.73m^2^)	66.2 [60.6] (38–111.4)	71.1 [71] (29.6–142)	74.6 [74.1] (20.9–161)
Changes in eGFR values (mL/min/1.73m^2^)^1^	-2.66 [-0.8] (-27.3 -14)	5.03 [4.8] (-24 -74.3)	1.33 [1.4] (-22 -36.4)
TP (g/dl)	6.56 [6.6] (5.1–7.9)	6.16 [6.4] (4.1–8.0) (*n* = 42)	6.43 [6.5] (3.6–7.7) (*n* = 32)
Alb (g/dl)	3.85 [3.85] (2.7–4.7)	3.61 [3.8] (1.5–4.4)	3.62 [3.8] (1.4–4.6)
Proteinuria (-/ ± /1 + /2 + /3 + /4 +)	(8/3/4/3/1/1)	(12/1/15/8/7/0)	(9/4/6/9/3/2)
Proteinuria (g/gCr)	1.33 [0.24] (0.04–9.55)	1.90 [0.6] (0.04–5.39)	2.46 [0.49] (0–20.6) (*n* = 32)
Thrombus event (%)	1 (5.0)	2 (4.7)	2 (6.1)

1 Changes in eGFR values (mL/min/1.73m^2^) are determined by subtracting the eGFR values at the biopsy from the eGFR values in the time of 1 year after renal biopsy

Most data value is shown as mean [median] (minimum–maximum). The Mann Whitney U and Fisher's exact tests were used for statistical analysis to compare NELL1-positive membranous glomerulonephritis to the other groups. (*p < 0.05, **p < 0.01, ***p < 0.001)

^#^ In the subgroup treated with Non-steroids, the NELL1-positive cases also exhibited significantly lower eGFR than Negative cases only at baseline, but not significantly after 1 year

^##^ In the subgroup treated with only steroids, the NELL1-positive cases also showed significantly lower eGFR than PLA2R1-positive cases both at baseline and after 1 year

## Discussion

Herein, we identified 24 cases of NELL1-positive MGN, comprising 9.3% (24/257) of all MGN cases, 10.9% (24/219) of primary MGN cases, and 23.2% (22/95) of PLA2R1-negative cases. Among primary MGN cases, the frequency of NELL1-associated MGN has been reported to be 2–2.5% in the US [7, 8] and 1.5% in Japan [23]. Among PLA2R1-negative primary MGN, the frequency of NELL1-associated MGN was 16% (34/210) in Europe and the US [6]. Among cases of primary MGN with both PLA2R1 and THSD7A negativity, the frequency of NELL1-associated MGN was 6.7% (3/45) in France [6], 5.1% (2/39) in Belgium [29], 34.9% (15/43) in China [30]. Therefore, the frequency of NELL1-positive MGN in this study was higher than that reported in Europe and the United States, whereas the results were similar to those of previous reports from China. A recent review showed NELL1 was the second most common antigen, accounting for approximately 10% [2]. It can be also highlighted that NELL1 is the second most common antigen followed by PLA2R1 in Japan.

Herein, we observed two cases that were dual-positive for NELL1 and PLA2R1. Among the NELL1-negative MGN cases, three were dual-positive for PLA2R1 and THSD7A. None of the patients tested were dual-positive for NELL1 and THSD7A. Two Chinese studies have reported four MGN cases that were dual-positive for NELL1 and PLA2R1 [30, 31]. Similarly, dual-positivity for both PLA2R1 and THSD7A has also been reported; however, its significance remains unclear [22]. Thus far, the clinicopathological significance of dual positivity in MGN remains unclear because of the limited number of cases. In this study, dual positivity was confirmed by immunohistochemistry only; a combined approach using immunohistochemical studies and western blot analysis [30] was not as serum samples for antibody tests were not obtained. Future studies with a larger number of patients, in addition to multiple diagnostic modalities of antibody detection, will be necessary to delineate the biological significance of the dual positivity of antibodies in MGN.

Histologically, NELL1-associated MGN commonly exhibits IgG1 predominant IgG subclass and segmental IgG patterns in IF [9]. In primary MGN, IgG subclasses generally demonstrate an IgG4 predominant distribution [32]. In addition, the incidence of segmental IF patterns among all patients with primary MGN was reported to be only 2.5% [33]. Thus, in NELL1-associated MGN, IgG1 dominant immunophenotype and segmental IgG patterns on IF may be suggestive of secondary MGN. Herein, the segmental distribution of GBM lesions was more prevalent in the NELL1-associated MGN group than in other groups, which is consistent with findings suggestive of secondary MGN. Regarding the IgG subclass, the frequency of IgG1 dominance was not significantly higher in the NELL1-associated MGN group. This finding may be a result of the low number of cases (*n* = 9) compared with the other two groups, and further studies with a higher number of cases are required.

Several other clinical and histological features observed herein may provide further insight into the characteristics of NELL1-associated MGN. First, patients in the NELL1-positive MGN group were older than those in the other two groups. Second, the segmental distribution of IgG may reflect the early phase of the disease. Huang et al. reported that IgG1 positivity was present in the early stages of MGN, which later switched to IgG4 positivity, suggesting that the predominant IgG subclass may switch as the disease progresses [34]. In addition, segmental MGN is reportedly more common in the early stages (Stage I-II) [33]. Herein, Ehrenreich–Churg stage I was significantly more prevalent among NELL1-positive MGN cases, which may have contributed to the segmental positivity and IgG1-dominant phenotype of the disease. However, Cui et al. reported that the switching of IgG subclasses was independent of the disease stage [35]. Additionally, the significantly shorter duration of the disease period of NELL1-positive MGN might have affected the results. Therefore, further studies are warranted to elucidate the association between the segmental distribution of IgG and the disease stage. Third, the observed higher prevalence of bucillamine administration in the NELL1-positive MGN group may provide mechanistic insights into this condition. Reportedly, bucillamine-related MGN exhibits segmental positivity on IF [36]. In a study by Miyazaki et al., of 10 patients with NELL1-associated MGN, 6 had RA, of whom 4 cases were drug-related and 2 showed segmental positivity [37]. These findings suggest that bucillamine may be associated with the pathogenesis of NELL1-positive MGN. In contrast, Takahashi-Kobayashi et al. reported a low frequency of bucillamine use among patients with NELL1-positive MGN. This observation suggests that the use of bucillamine is more prevalent in older patients and may potentially be influenced by case sampling [38]. Therefore, the association between bucillamine administration and NELL1-positive MGN needs to be confirmed in future studies.

Herein, no clear differences in prognoses were observed between the groups. Additionally, although no significant differences were observed, there were fewer cases of non-remission and more cases of complete and partial remission in the NELL1-positive MGN group than in the PLA2R1-positive MGN group. Both the mean and median changes in eGFR after 1 year showed improvements in the NELL1-positive MGN group. However, NELL1-positive cases showed lower eGFR values after 1 year than PLA2R1-positive and Negative cases. Additionally, in the subgroup treated with non-steroids, NELL1-positive cases exhibited significantly lower eGFR than Negative cases at baseline; however, this was not significant after 1 year. In the subgroup treated with only steroids, patients with NELL1-positive MGN also exhibited significantly lower eGFR than patients with PLA2R1-positive MGN, both at baseline and after 1 year. These differences may be attributed to the lower baseline eGFR in NELL1-positive cases. Pathologically, extracapillary hypercellularity, segmental sclerosis, IFTA, and arteriolar hyalinosis have been identified as poor prognostic factors for MGN [39]. Among these, IFTA was a poor prognostic factor [40]. Additionally, a previous report indicated that patients with an advanced histological stage (Ehrenreich–Churg stage III or stage IV) have poorer prognoses [41]. In our study, patients in the NELL1-positive MGN group exhibited a lower prevalence of segmental sclerosis and a greater prevalence of histological stage I than those in the PLA2R1-positive MGN and Negative groups. These findings may be associated with better prognosis, more cases of remission, and less decline in renal function after treatment of NELL1-positive MGN. However, one report indicated that the Ehrenreich–Churg stage is not related to prognosis [40]. Additionally, the IFTA rate and total renal chronicity score, which are indicative of a poor prognosis, were higher in NELL1-positive MGN cases in the present study. Therefore, a stratified analysis with a sufficient number of cases is necessary for multivariate analysis.

The present study has some limitations. It was a retrospective study, and selection bias in the inclusion of the cases could not be ruled out. Additionally, the number of patients in each group was small and the number of studies was insufficient for stratification. In particular, due to the small number of THSD7A-positive cases, its clinical and pathological characteristics could not be compared with those of NELL1-positive MGN cases. While NELL1 is associated with malignancy [9], the NELL1-positive MGN patients with complicated malignancy in this study were not significantly more common, indicating another limitation related to the small sample size. We also have several limitations in the pathological assessment. Different immunostaining approach (i.e., immunofluorescence for PLAR1 and IHC for NELL1 and THSD7A) might have affected the comparison of the clinicopathological characteristics, especially the positivity prevalence of the indicated antigens. Regarding the association between the disease stage by EM and the period from disease onset to the biopsy, it was difficult to identify the exact period because the disease onset of MGN was generally vague. Regarding clinical characteristics, the degree of remission (complete remission, partial remission, and no remission) after treatment differed according to the treatment group (non-steroid, steroid-alone, and steroid plus immunosuppressive agent). Further large-scale studies are needed to confirm these findings.

In summary, this study revealed that NELL1-positive MGN cases comprised 9.3% (24/257) of total MGN cases and 10.9% (24/219) of primary MGN cases. Histologically, NELL1-positive MGN have tended to show segmental distributions in both LM and IF. The segmental distribution and IgG1-dominant IgG subclass may be linked to bucillamine-related MGN and the early stages of EM. Future studies with larger numbers of enrolled patients are needed to delineate the prognosis of NELL1-positive MGN.

## Data Availability

The datasets generated and/or analyzed in the current study are available from the corresponding author upon reasonable request.
